# Quantifying Cutting and Wearing Behaviors of TiN- and CrN-Coated AISI 1070 Steel

**DOI:** 10.3390/s8116984

**Published:** 2008-11-05

**Authors:** Ahmet Cakan, Vedat Ozkaner, Mustafa M. Yildirim

**Affiliations:** 1 Department of Mechanical Engineering, Abant Izzet Baysal University, Bolu, Turkey; 2 Department of Electirical-Electronic Engineering, Mustafa Kemal University, Hatay, Turkey; 3 Faculty of Technical Education, Department of Metallurgy, University of Firat, Elazig, Turkey

**Keywords:** TiN-coating, CrN-coating, cutting performance, tool wear on-line monitoring, cutting force

## Abstract

Hard coatings such as titanium nitride (TiN) and chromium nitride (CrN) are widely used in cutting and forming tools against wear and corrosion. In the present study, hard coating films were deposited onto AISI 1070 steels by a cathodic arc evaporation plating (CAVP) technique. These samples were subjected to wear in a conventional lathe for investigating the tribological behaviour of coating structure, and prenitrided subsurface composition was characterized using scanning electron microscopy (SEM), line scan analyses and X-ray diffraction (XRD). The wear properties of TiN- and CrN-coated samples were determined using an on-line monitoring system. The results show that TiN-coated samples demonstrate higher wear resistance than CrN-coated samples.

## 1. Introduction

Thin film coating is applied to improve the wear resistance of substrates owing to its hardness and high mechanical strength. Applications of thin films are steadily increasing in various industrial fields [1]. Among many hard coatings, PVD (physical vapor deposition) TiN (titanium nitride), TiC (titanium carbide) and CrN (chromium nitride) are the most widely used ceramic coatings in cutting tools due to their high hardness and wear resistance, low coefficient of friction [2], high temperature strength, chemical stability [3], and corrosion resistance [4]. However, application of PVD hard coatings to the substrate materials cannot guarantee the optimal tribological performance without pretreatment of the substrate materials due to plastic deformation of the substrate, which results in eventual coating failure. The surface treatment provides hardening and increases the load support effect for the substrate [5].

Many approaches aim to enhance the load bearing capacity of the coated surfaces and improve the adhesion of the coatings such as deposition of an interlayer material or compound prior to the coating or the treatment of the surface by nitriding [6-9], carburizing, borizing, nitrocarburizing and so on. However, in terms of the cost and complexity, nitriding or carburising is a better candidate than the other methods. Previous research showed the beneficial effects of nitriding steel before PVD coating. For example, nitriding was found to increase the lifetime of cutting tools made of tool steel [10].

The aim of this study is to investigate the possibility of using PVD hard coatings such as TiN and CrN on the medium carbon substrates, which are typically used in cutting tool applications. Therefore, TiN and CrN hard coatings were deposited on prenitrided medium carbon steel (AISI 1070). The steel substrates were nitrided in molten salt bath before the coating process. On-line monitoring system was used to investigate the wear resistance of CrN and TiN coatings, which were applied on AISI 1070 tool steel using the cathodic arc evaporation process technique. Experiments were carried out in a conventional lathe and the following parameters have been measured: the wear volume, cutting force, feed force, and thrust force of the counter material. The performance of the prenitrided TiN- and CrN-coated cutting tools was comparatively studied.

## 2. Experimental Procedure

### 2.1. Sample Preparation

**Figure 1.**
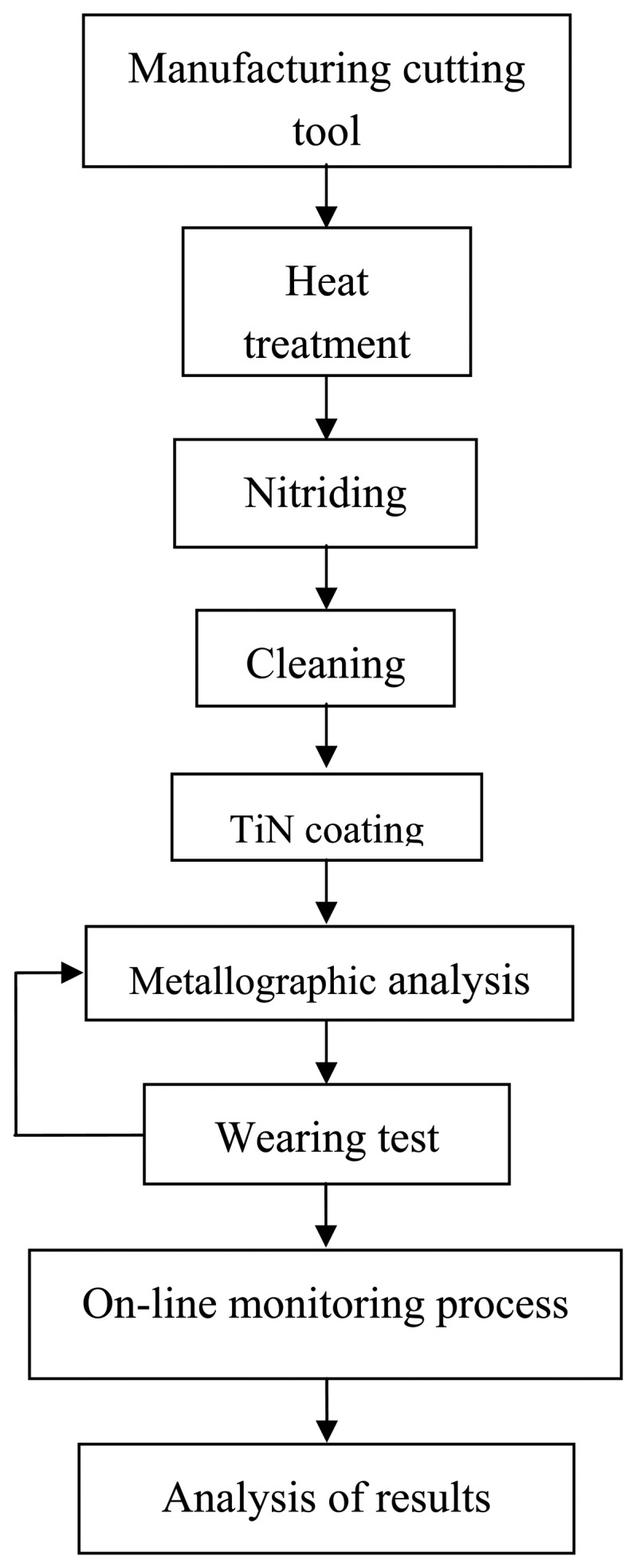
Experimental process.

Substrate samples with the size of 12mm×12mm×100mm were cut from medium carbon steel AISI 1070. The samples were heat-treated in a furnace at a temperature of 760 °C for a period of 20 minutes and then they were quenched starting from the tip until the room temperature. The final hardness of the tool samples was about 62 HRC. All the samples were tempered at 180°C for a period of 40 min with the following composition (wt%) 0.794% C, 0.0229% Si, 0.023% S, 0.016% P, 0.3487% Mn, 0.0396% Ni, 0.0133% Cr, 0.0016 Mo, 0.0012% V, 0.02% Cu, 0.0016% Ti, 0.0011% Sn, and 0.0273% Al. These samples were subsequently grounded, hardened, nitrided, polished and cleaned before the coating. The steps for the sample preparation are given in Fig.1.

### 2.2. Salt Bath Nitriding

To obtain a nitrided layer, nitriding was used as the thermo-chemical surface treatment method, which improves the wear resistance of cutting tools. The cutting tools made of steel grade AISI 1070 were nitrided in a molten salt bath (CN) at about 570°C for 2 h. It is reported that, in a conventional process with high nitriding potential, partially compound zone of about 30 μm thick was formed [11]. The inner part is reported to be a γ'–Fe_4_N, ε-Fe_2_-_3_(NC), or mixed ε-γ' phase [11]. Conventional molten salt bath can be used to achieve the above described structure.

### 2.3. Coating

TiN and CrN coatings were deposited onto prenitrided cutting tools by using cathodic arc evaporation technique (CAVP) as the PVD coating method. Prior to coating, the cutting tool samples were polished by mechanical methods to the surface roughness of R_a_ ~ 0.1 μm. Then these samples were cleaned in an ultrasonic bath using basic alkali cleaner. The samples were vertically fixtured on a continuously rotating planetary holder inside the vacuum chamber. The purity of N_2_ gas used was 99.998% and that of the Ti target was 99.6 %. Prior to deposition, chamber was further cleaned by glow discharge by using high purity N_2_ gas with a partial pressure of 15-20 mtorr and -1000 V substrate bias voltage. During TiN deposition, the chamber pressure was maintained at 3 × 10^-3^ torr by introducing nitrogen. A negative bias voltage of -200 V was applied to the substrate during deposition. During CrN deposition, the chamber pressure was maintained at 7 × 10^-3^ torr, while a negative bias voltage of 100 V was applied to the substrate. Deposition period of TiN and CrN was 45 min and 30 min, respectively. The thickness of TiN film reached was approximately 1.3 μm, while that of CrN film was approximately 3 μm.

### 2.4. Cutting Condition

Cutting tests were carried out on a 1.2 kW conventional lathe machine under dry cutting conditions. The tools were tested at the spindle speeds of 190, 240 and 320 rpm (at cutting speeds of 23.89 m min^-1^, 30.18 m min^-1^ and 41.12 m min^-1^) with a feed rate 0.081 mm rev^-1^. A depth of cut of 0.8 mm was used for shape manufacturing and was kept constant throughout the tests.

### 2.5. Workpiece Materials

The cutting performance tests were performed on DIN 9SMnPb36 steel bars. Based on the carbon steel standard of AISI-SAE, its composition is (wt%) 0.15% C, 0.05% Si, 1.50% Mn, 0.100% P, 0.37% S, and 0.15% Pb. The workpiece material used has a dimension of 200 mm of length and 40 mm of diameter.

### 2.6. On-line Monitoring of Tool Wear

Many systems were developed for indirectly detecting tool wear in turning and milling operations [12-16]. In the present study, the amount of flank wear on a turning tool is indirectly determined, without interrupting the machining operation, by monitoring changes in the workpiece diameter using a photo electronic sensor [17-19]. The sensor consists of a bifurcated optical fibre, a laser diode (650 nm 20mW Visible red) as the light source and a photodiode (IPL10530DAL) as the detector, which has amplification circuit. The flow chart of the experimental process is shown in Fig. 1.

**Figure 2.**
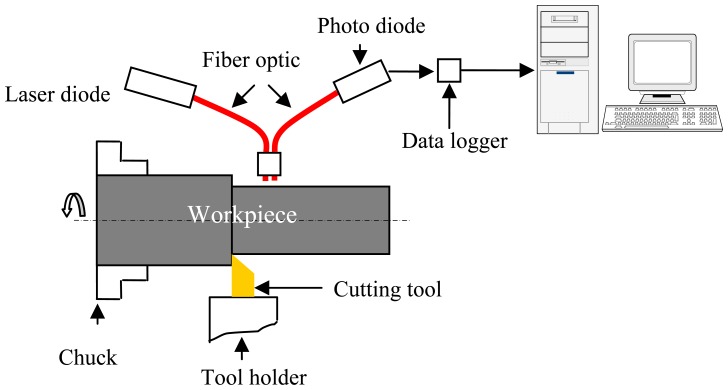
Schematic diagram of the experimental set-up.

A schematic diagram of the experimental set-up is shown in Fig. 2. The optical fiber is held in front of the workpiece diametrically opposite to the cutting tool. The optical fiber is fixed in such a way that it lags the cutting tip by a certain distance and its axis intersects the axis of rotation of the workpiece. This is to ensure that the laser beam is incident on and reflected back by the freshly produced work surface. The optical fiber used has a core of 3 mm diameter. The core fibers are coupled with brass sleeves at the ends. A 20 mW Laser diode is used as the light source. The power of the laser beam should be such that it does not drive the photodiode into the saturation region and yet provides signals of sufficient amplitude. It is necessary to ensure that the laser beam produced is of a constant intensity throughout the experiments. A brass coupling is used to connect the source fibers of the sensor to the laser diode. This is to ensure that there is no relative movement between the source fibers and the laser diode.

The sensing fibers are connected to the photodiode using another brass coupling to avoid any relative movement between them. This set-up ensures that any change in the output signal is only because of the change in the intensity of the reflected light. The cable carrying the output signal from the photodiode is connected to a data logger. The data were recorded every second by the data logger and were transferred to a PC by using serial port.

### 2.7. The Cutting Force Measurements

**Figure 3.**
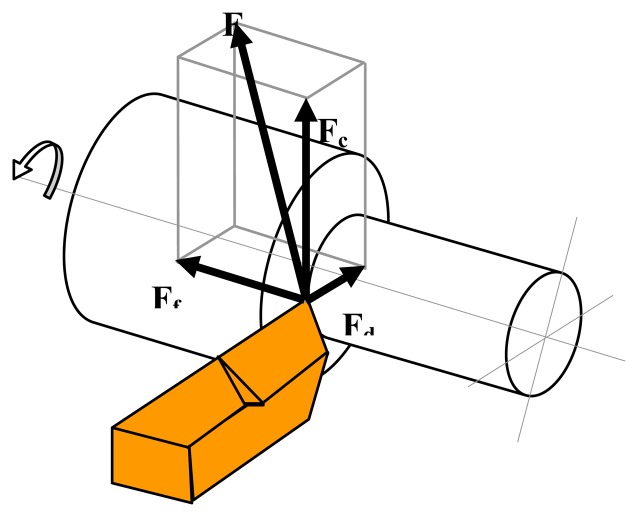
Vector orientation.

When a cylindrical workpiece is turned, the cutting force **F**, as shown in Eq. (1), may be divided into three vector force components (see Fig. 3 for vector orientation):

(1) $$F=F_{c}+F_{f}+F_{d}$$

Where ***F****_c_* is the tangential component, ***F****_f_* is the feed force component and ***F****_d_* is the radial force. Force measurement is essential to understanding the cutting mechanism such as the effect of cutting variables on the cutting force, the machinability of the work piece, the process of chip formation and tool wear. The need for measuring the cutting forces is due to the fact that they change with the progress of tool wear. Therefore, tool wear can be determined by monitoring the force variation. The cutting force measurement is a good indicator in detecting tool wear. For this purpose, many dynamometers have been developed [20-22].

During the experiments, these three forces on the cutting tool were measured. These orthogonal components of the force were measured by using the strain gauge based on a three component-cutting force dynamometer [20]. The output signals obtained from the dynamometer for the forces were amplified by using three strain gauge amplifiers. The cables carrying the output signal from these amplifiers were connected to the data logger. The data were logged at 1 s interval and were transferred to the PC once the measurement was complete.

## 3. Results and Discussion

Turning experiments were performed with prenitrided medium carbon steel cutting tools coated with CrN and TiN. CrN- and TiN-coated tools were tested under the same cutting conditions. The geometrical configurations conform to Turkish Standard (TS 9044). The material of workpiece was DIN 9SMnPb36 steel. The dimensions of the cylindrical workpiece were 40 mm in diameter and 200 mm in length. The cutting parameters of the lathe were as follows: feeding rate was 0.081 mm rev^-1^, spindle speeds were 190, 240 and 320 rpm (cutting speeds were 23.89 m min^-1^, 30.18 m min^-1^, and 41.12 m min^-1^), and depth of cut was 0.8 mm. The total length of cut was 150 mm. No cutting fluid was added throughout the turning experiment.

**Figure 4.**
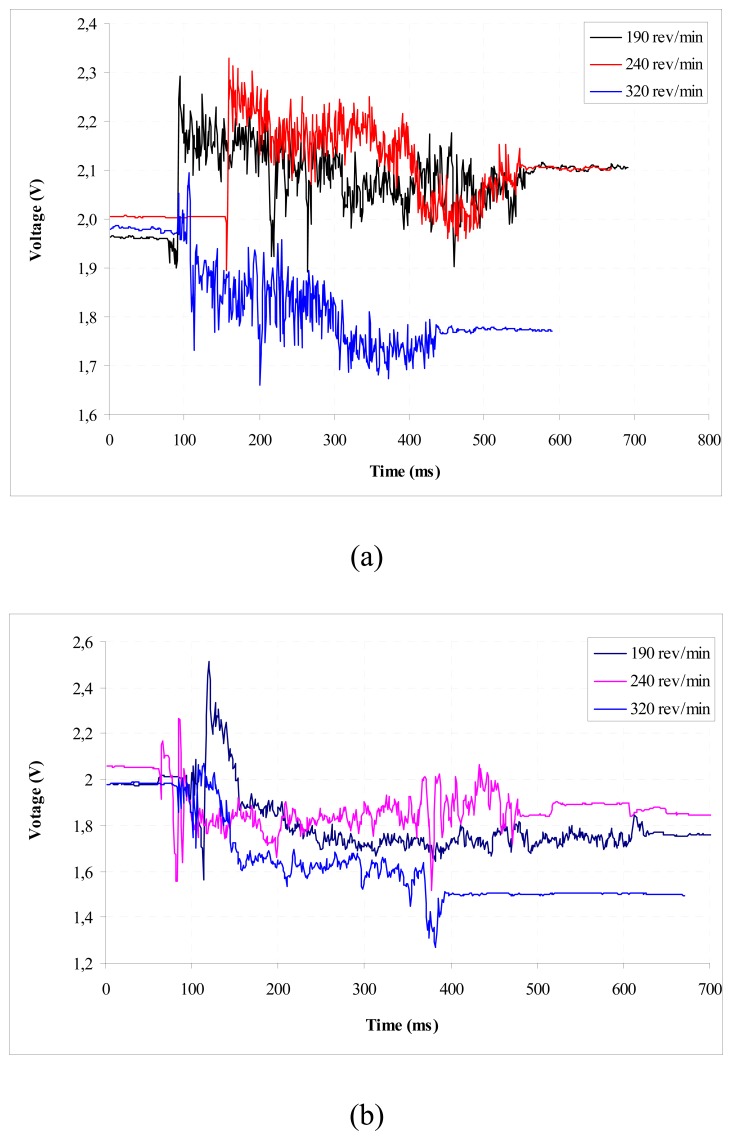
Comparison of wear properties at different rotational speeds at a feed speed of 0.081 mm rev^-1^ for (a) TiN-coated and (b) CrN-coated cutting tools.

The workpiece was held in a three-jaw chuck and supported in the centre at its tailstock. The other cutting parameters were set as required for the particular experiments. After machining with a fresh tool for about 15 mm length on the workpieces, the process was stopped and initial gap between the sensor and the workpiece was adjusted to 2.3 mm. The machining operation was then continued for the rest of the workpiece. During machining, the sensor output was continuously recorded. The metal cutting performance of coated tools is a function of substrate, coating, and geometry of the cutting edge. For the given metal cutting test, we kept the substrate and the geometry constant. Therefore, differences in the tool wear and cutting forces observed during the test should be attributed only to the differences in the properties of the coated materials and their adhesion to the substrate. The results are summarized in Fig 4.

### 3.1. Voltage Variation

In most of the experiments, an initial increase in the voltage was observed in contrary to an expected decrease. This phenomenon was also observed and reported by other studies [17,18,23]. The amount of light reflected from the workpiece surface and detected by the photodiode is a function of two parameters: firstly, the distance between the sensor and the workpiece surface, and secondly reflectance of the workpiece. A small nose radius was formed at the sharp cutting tip of the tool due to flank wear sometime after the machining operation was started with a new tool. This leads to a better surface quality of the workpiece and thus a higher reflectance. The gap between the workpieces and the sensor reduces due to the flank wear and notching on the cutting tool, which leads to further drop in the voltage signal during the machining operation. This phenomenon was also observed and reported elsewhere [17].

### 3.2. Wear

Turning tests have been conducted in order to study the wear mechanism of the CrN and TiN coatings during the cutting process. The relationship between the flank wear and the cutting length of the CrN- and TiN-coated cutting tools is shown in Fig. 4. Our results indicated that the TiN coating had a better wear resistance than CrN coating. The turning tests conducted at higher cutting speeds showed that the flank wear increased with the increasing cutting speed. Also in higher speeds, TiN-coated cutting tool had a higher wear resistance than CrN-coated one as can be seen in Fig. 4.

### 3.3. Adhesion

**Figure 5.**
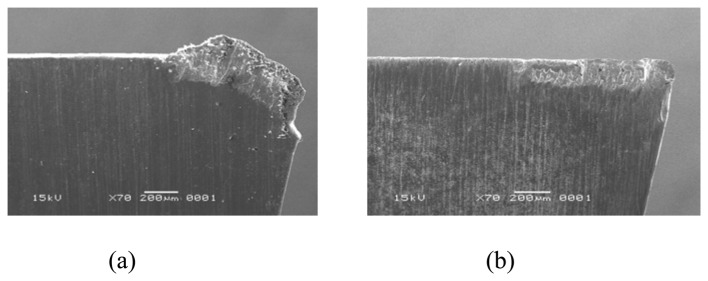
Flank wear of the coated samples (V_c_ = 41.12 m min^-1^): (a) CrN and (b) TiN.

As the TiN and CrN-coated tools advance, the turning workpiece material continues to adhere to the cutting edge and flank face. Wear progresses as the adhered layers, TiN and CrN coatings and even the prenitrided high carbon steel substrate are carried by the flowing chips. The wear phenomenon taking place in TiN- and CrN-coated prenitrided high carbon steel after 150 mm of cutting length appears in Fig.5. One can see a large amount of adhered layer, which is the transferred DIN 9SMnPb36 steel material, covered in the flank face. As the chromium content of the coating material increases, the adhesion phenomenon in the dry turning becomes more accentuated [24, 25]. Therefore, the CrN-coated tool had a larger flank wear than TiN-coated one.

Our results indicated that the CrN coatings had strong chemical affinity to the DIN 9SMnPb36 steel during the turning process. According to the phase diagrams, Fe and Cr have completely unlimited mutual solid solubility with each other over a temperature of 800 °C [26]. This was the reason for the adhesion. In the case of the CrN-coated insert, a strong bonding force between the adhered DIN 9SMnPb36 steel material and the CrN coating was formed, due to high chemical affinity between Fe and Cr, which occurred at the temperature induced during turning. As the size of the adhered DIN 9SMnPb36 steel material reached a critical value, it was detached, and thus, so were the underlying CrN coating and small pieces of prenitrided high carbon steel substrate. Thus, the prenitrided high carbon steel substrate was severely fractured by the adhesion. Fig. 5(a) is the SEM micrograph of the wear on the CrN-coated prenitrided medium carbon steel after 150 mm cutting length of the DIN 9SMnPb36 steel material. The worn surface exhibits very smooth topography and good conformity with the chip flow pattern. Such smoothly worn surface topography is characteristic of abrasive wear. It is inferred in the present article that the CrN and TiN layers are worn off by abrasion process.

### 3.4. Cutting Force

**Figure 6.**
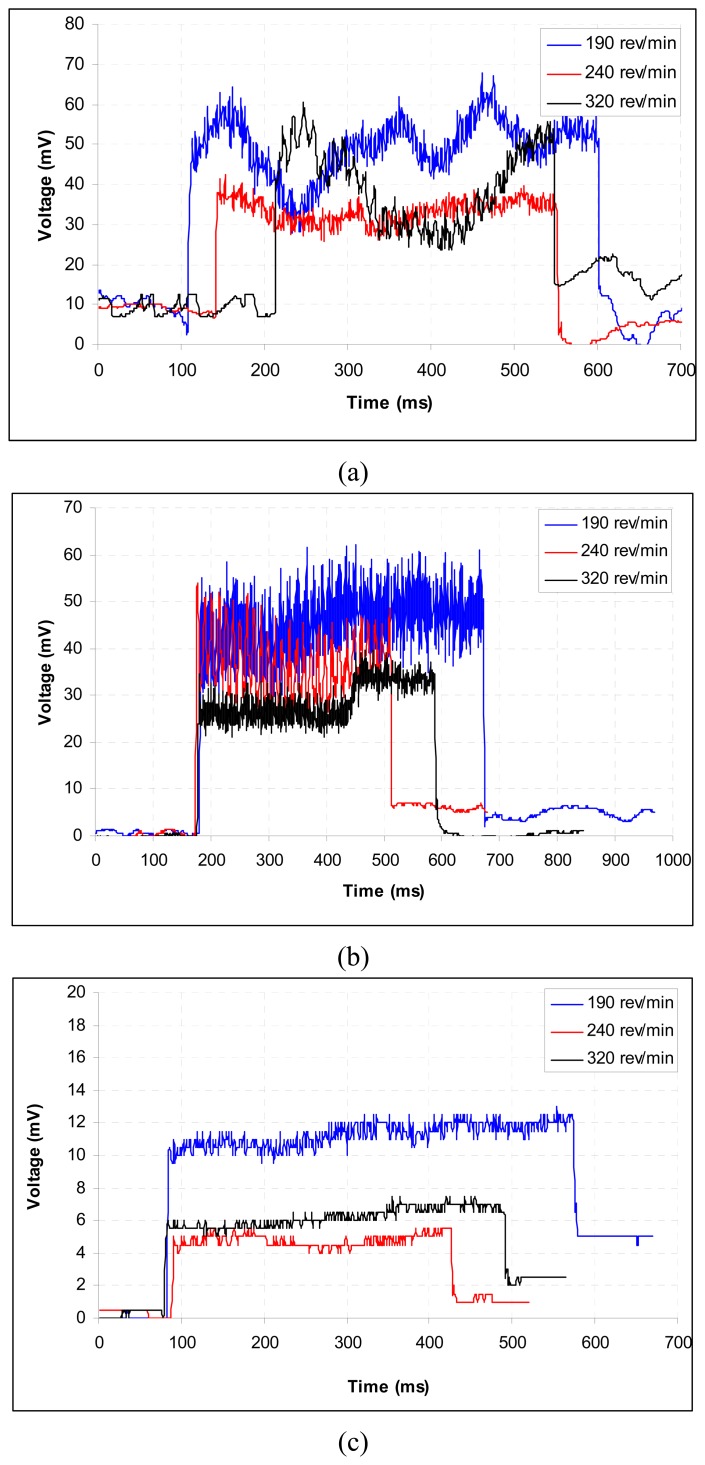
Comparison of cutting forces at different rotational speeds at a feed speed of 0.081 mm rev^-1^ for TiN-coated cutting tools: (a) cutting force: *y* = -0.501*x* + 1.0698 (*R*^2^ = 0.9998; *P* < 0.001), (b) feed force: *y* = 80.26*x* + 124.9 (*R*^2^ = 0.9995; *P* < 0.001) and (c) radial force: *y* = 7.94*x* + 128.5 (*R*^2^ = 0.9993; *P* < 0.001). *y* and *x* values are in kN and in mV, respectively.

**Figure 7.**
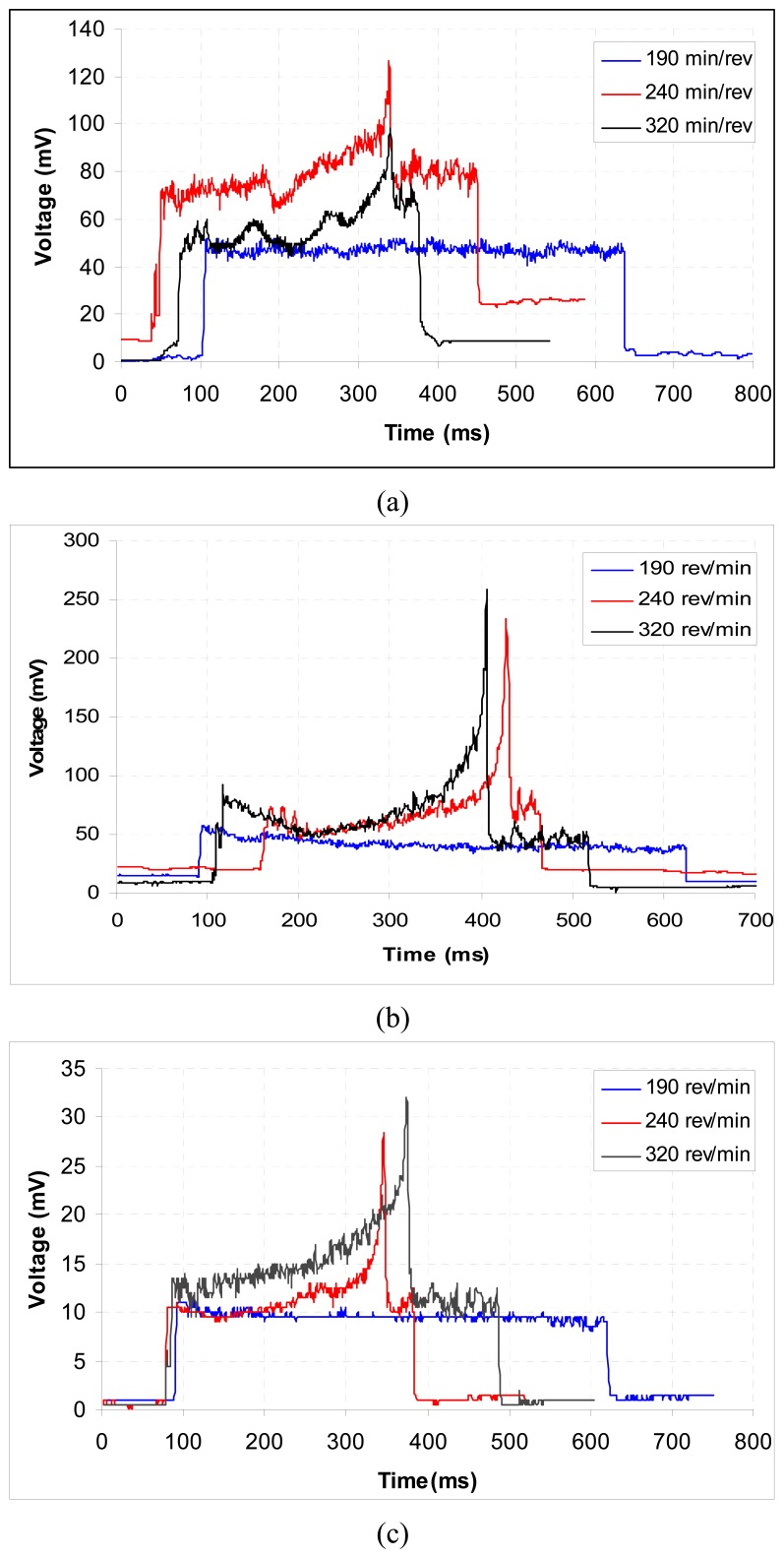
Comparison of cutting forces at different rotational speeds at a feed speed of 0.081 mm rev^-1^ for CrN-coated cutting tools: (a) cutting force: *y* = -0.501*x* + 1.0698 (*R*^2^ = 0.9998; *P* < 0.001), (b) feed force: *y* = 80.26*x* + 124.9 (*R*^2^ = 0.9995; *P* < 0.001) and (c) radial force: *y* = 7.94*x* + 128.5 (*R*^2^ = 0.9993; *P* < 0.001). *y* and *x* values are in kN and in mV, respectively.

The cutting, feed and radial forces versus cutting speed relationships for the experimental tests are shown in Figs. 6 and 7. Figures also show the effect of different coatings and cutting speeds. For the CrN-coated cutting tools, the cutting force increased when the cutting speed was increased from low to medium.

The force, however, decreased when the cutting speed was at the maximum level. The feed and radial forces were about twice lower than the cutting force. Both other forces, however, decreased with and increase in cutting speed. For the TiN-coated cutting tools, all the forces decreased with an increase in cutting speed. The correlations between the voltage (mV) and force (kN) for the variables of cutting, feed and radial forces were derived from these best-fit equations as shown in Figs. 6 and 7.

### 3.5. Microstructural Aspects

**Figure 8.**
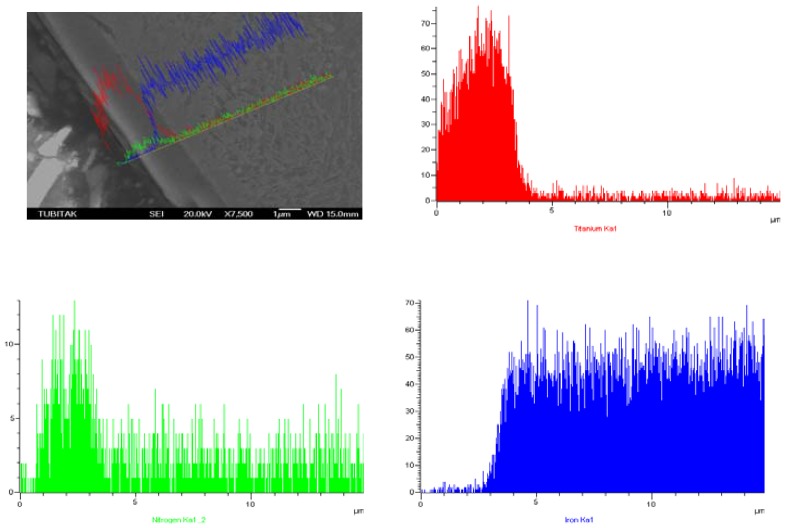
EDS line-scan analysis of TiN-coated sample.

**Figure 9.**
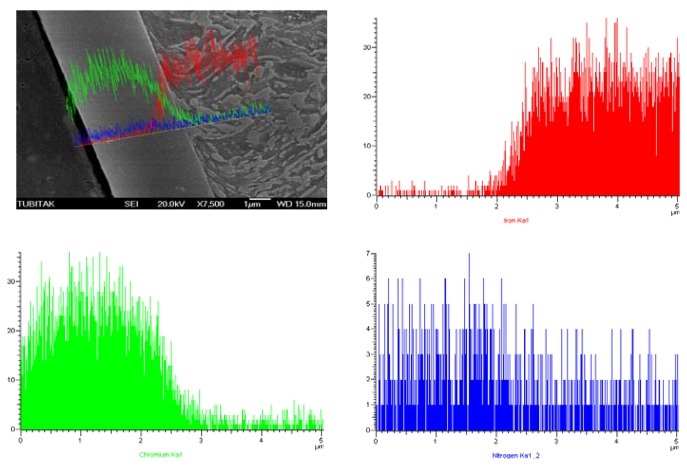
EDS line-scan analysis of CrN-coated sample.

SEM Line Scan analysis of cross-section of prenitrided high carbon steel coated with TiN and CrN is shown in Figs. 8 and 9, respectively.

Variation in the contents of Ti, nitrogen (N) and iron (Fe) across the coated region is given in Fig. 8, and variation in those of Cr, N and Fe is given in Fig. 9, starting from the cladding layer towards the substrate, determined using the line scan analysis technique.

As seen in Fig. 8, Ti is present substantially in the outer region of the cladding layer, and its presence decreases sharply on the sub-surface line between the white layer and the cladding layer. Hardly any Ti exists within the matrix. It was observed that N heavily existed in the prenitrided surface. However, it was also observed that N existed in an evenly diffused way in the white layer and within the matrix itself. A small amount of Fe seemed to diffuse to the cladding region. Starting from the white layer, its ratio increased rapidly towards the substrate (Fig. 8). The reason for the sharp increase in the amounts of Ti and N from the outer to the inner region can be attributed to the relatively low temperature (180 °C) used, and the short period of the treatment time.

**Figure 10.**
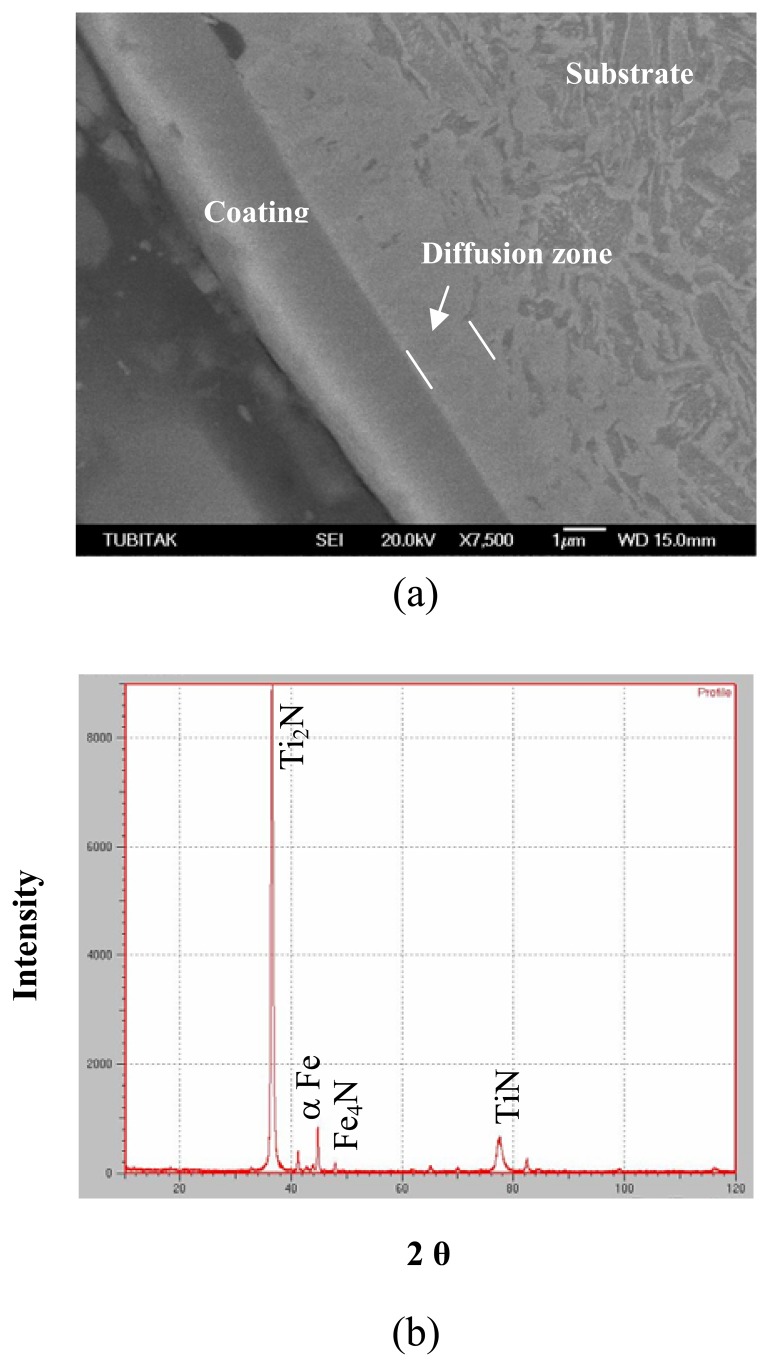
TiN-coated specimen: (a) SEM micrograph showing the microstructure across the cross-section and (b) XRD spectrum.

**Figure 11.**
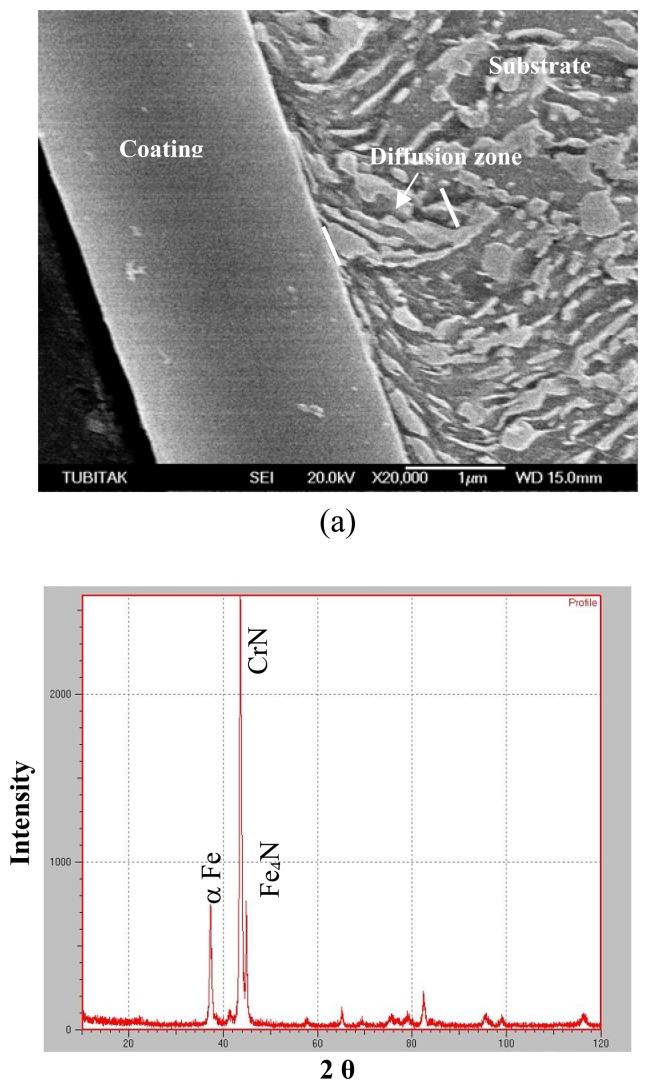
CrN-coated specimen: (a) SEM micrograph showing the microstructure across the cross-section and (b) XRD spectrum.

Fig. 10 presents the XRD spectrum of cross-section of prenitrided high carbon steel coated with TiN. From the diffraction peaks of cross-section, Ti_2_N, TiN, α-Fe, and γ^'^-Fe_4_N phases are present in the coated layer of TiN-coated specimen. Fig. 11 exhibits X-ray diffraction spectrum of cross-section of prenitrided high carbon steel coated with CrN. It can be seen that CrN, α-Fe, and γ^'^-Fe_4_N phases are present in the coated layer of CrN-coated specimen.

## 4. Conclusions

As metal cutting process is quite complicated, it is very difficult to determine mechanisms in which cutting parameters affect tool wear and cutting forces. We used an optoelectronic sensor system to monitor the tool wear without interrupting machining process. We based the provision of a reliable and sensitive technique for on-line monitoring of the tool wear on the basic idea of measuring the flank wear and notching on the tool indirectly by correlating it with the workpiece dimension. In the present work, thorough experimental investigations were conducted in order to study the effects of prenitrided substrate and surface mechanical treatments on the coating adhesion and cutting performance of TiN-and CrN-coated cutting tools. Accordingly, appropriate turning experiments were conducted at different cutting conditions to determine the amount of tool wear taking place on the flank.

Our experimental results revealed that prenitrided substrate and surface treatment enhanced coating adhesion properties and led to an increased turning performance of cutting inserts in the case of TiN-coating. This is also verified by observed changes in the cutting forces during the cutting process. The substrate on CrN plating worsened the adhesion strength, as well as the tool cutting performance.
